# A Portable, Low‐Cost Spectrophotometer for Absorbance and Selected‐Wavelength Fluorescence Measurements

**DOI:** 10.1155/jamc/9371485

**Published:** 2026-10-28

**Authors:** Sanam Pudasaini, Prakash Dangal, Rajesh Singh Rathore, Beni B. Dangi, Vijay Singh Chhetri

**Affiliations:** ^1^ Department of Chemistry Florida Agricultural and Mechanical University Tallahassee 32307 Florida USA famu.edu; ^2^ Food Science Program College of Agriculture and Food Sciences Florida Agricultural and Mechanical University Tallahassee 32307 Florida USA famu.edu; ^3^ School of the Environment Florida Agricultural and Mechanical University Tallahassee 32307 Florida USA famu.edu

**Keywords:** absorbance, analytical chemistry, fluorescence measurement, spectrophotometer

## Abstract

We present a portable, low‐cost, modular spectrophotometric platform designed for wavelength‐selective optical measurements using interchangeable visible LEDs, an AS7343 multichannel spectral sensor, and Arduino‐based data acquisition. The system was developed to provide a compact alternative for optical absorbance and fluorescence measurements while maintaining performance comparable to conventional laboratory instrumentation. The device was evaluated using aqueous solutions of copper (II) sulfate (CuSO_4_) and potassium permanganate (KMnO_4_), and results were benchmarked against a standard UV–Vis spectrophotometer. The system demonstrated concentration‐dependent absorbance responses consistent with the Beer–Lambert law, with strong calibration linearity (*R* ^2^ = 0.99 for CuSO_4_ and *R* ^2^ = 0.97 for KMnO_4_). In addition, the platform was adapted for fluorescence measurements of Rhodamine B via a simple reconfiguration to a 90° excitation–emission geometry. The fluorescence response showed linear behavior over the 0–4‐μM range, yielding an *R* ^2^ value of 0.97. The results indicate that the proposed system provides reliable optical measurements for both absorbance and fluorescence modalities, offering a flexible and cost‐effective alternative for analytical spectrophotometry applications in resource‐limited laboratory settings.

## 1. Introduction

Spectrophotometry is a fundamental analytical technique widely used in chemistry for the quantitative determination of chemical species, reaction monitoring, and characterization of materials [1–3]. Its operation is based on the Beer–Lambert law, a fundamental principle in analytical chemistry that describes the relationship between absorbance and analyte concentration. This law underpins spectrophotometric measurements and provides the basis for quantitative analysis through the relationship between absorbance and analyte concentration. However, conventional spectrophotometers are often expensive, bulky, and limited in accessibility, particularly in resource‐constrained laboratories.

To address these limitations, a range of alternative low‐cost and portable spectrophotometric approaches have been explored, including smartphone‐based detectors, simple LED–photodiode systems, and open‐source microcontroller platforms [4–9]. Smartphone spectrophotometers leverage built‐in cameras and apps for colorimetric analysis, offering portability but often suffering from variability in camera sensitivity and limited spectral specificity [10–12]. LED–photodiode systems provide simplicity and low cost but are typically restricted to single‐wavelength measurements and lack spectral resolution [7]. More advanced devices have incorporated compact spectral sensors and microcontrollers, enabling multiwavelength detection while maintaining affordability and ease of use [5, 13, 14]. Previously, our group developed a portable fluorescence spectroscopy setup based on mini lasers, a portable spectrometer, and a laptop computer for demonstrating spectroscopy and fluorescence analysis, with an estimated system cost of approximately $3000 [15]. The instrument enabled acquisition and analysis of fluorescence spectra from inorganic and organic samples, demonstrating the feasibility of portable fluorescence measurements. A comparison of the present system with representative low‐cost and portable spectrophotometric platforms, considering cost, dimensions, spectral characteristics, number of wavelength channels, measurement modes, and applications, is provided in Table S2. In the present work, we extend this approach by developing a portable single‐wavelength LED‐based absorbance and fluorescence measurement system with a total cost of around $50. Previous studies have demonstrated compact analytical systems capable of both absorbance and fluorescence measurements using LED‐based excitation and optical detectors. For example, Malecha developed an LTCC‐based microfluidic platform integrating LEDs, optical detectors, and optical fibers for absorbance and fluorescence measurements. In contrast, the present work employs a simplified cuvette‐based architecture using commercially available optoelectronic components and a multichannel spectral sensor, eliminating the need for specialized microfluidic fabrication while allowing reconfiguration between absorbance and fluorescence measurement modes [16].

In this context, the objective of the present work was to develop a low‐cost, portable, modular, and reconfigurable spectrophotometric system capable of performing both absorbance and selected‐wavelength fluorescence measurements using readily available optoelectronic components. The system utilizes interchangeable visible‐light LEDs as excitation sources and a multichannel color sensor for detection, allowing measurements at selected wavelengths relevant to specific chemical analyses (Figure 1A) [12, 17]. The actual system is shown in Figure 1B. By integrating microcontroller‐based data acquisition with inter‐integrated circuit (I^2^C) communication and serial data output, the platform enables direct digital control and readout of spectrophotometric measurements. This architecture supports flexible instrumentation by allowing precise control and monitoring of measurement parameters such as wavelength selection, optical path length, and signal processing for absorbance determination. As a result, the system provides a compact and cost‐efficient analytical tool that combines hardware modularity with digital data handling, facilitating reliable optical measurements in laboratory and field environments. The contribution of this work is, therefore, not the use of individual low‐cost optoelectronic components, which have been widely reported, but their integration into a compact, modular, and reconfigurable multichannel platform capable of performing both absorbance and fluorescence measurements using a common optical architecture. In addition, the performance of the developed system is quantitatively evaluated and compared with conventional spectrophotometric measurements to establish its analytical capability.

**FIGURE 1:**
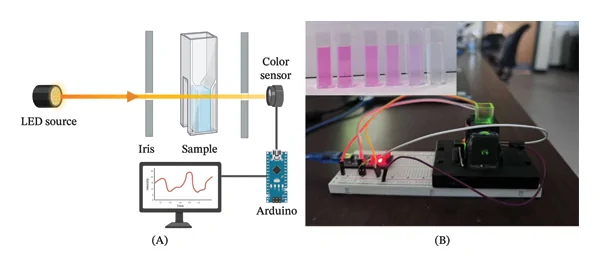
(A) Schematic of the system for absorbance measurement. (B) Photograph of the portable fluorescence measurement system built on a standard breadboard platform (approximately 17 × 6 cm), demonstrating its compact design. The system can be powered using a standard 9‐ or 12‐V battery, making it suitable for portable teaching and learning applications.

## 2. Materials and Methods

### 2.1. Chemicals and Reagents

CuSO_4_.5H_2_0 (AC423615000, Thermo Scientific Chemicals) and KMnO_4_ (AA1430722, Thermo Scientific Chemicals) were used as the model analytes for spectrophotometric measurements. Aqueous solutions of varying concentrations were prepared using deionized water as the solvent. All solutions were prepared using analytical‐grade reagents and were used without further purification. Spectral measurements were carried out using a custom‐built, low‐cost spectrophotometric system equipped with interchangeable visible LEDs, with excitation at 630 nm (LED630L, Thorlabs, USA) and detection at 640 nm for CuSO_4_, and excitation at 528 nm (LED528EHP, Thorlabs, USA) and detection at 515 nm for KMnO4. For fluorescence measurement, Rhodamine B (419000010, Thermo Scientific Chemicals) was used.

### 2.2. Instrumentation

The experimental setup consisted of a visible‐light optical measurement system integrating a multichannel color sensor (AS7343, SparkFun Electronics, USA) with interchangeable LEDs as illumination sources (Figure 2). LEDs of specific wavelengths were selected based on the analytical requirements of the target chemical species, with the flexibility to replace LEDs to match desired absorption bands within the visible spectrum, consistent with the operational range of the color sensor. Typical spectral intensity distributions for both LEDs used in this study are presented in Figures S1 and S2. The LEDs were powered using the 5 V output of the Arduino Nano through a 1‐kΩ series resistor to limit the forward current. The selected operating voltage was within the manufacturer’s specified operating conditions, and the same 5 V supply was maintained for each LED type throughout the experiments to provide consistent excitation conditions. The emitted light was directed through the sample contained in a cuvette with a fixed optical path length of 10 mm, after which the transmitted light intensity was detected by the color sensor. The cuvette holder in the DIY was obtained from scrap and can be built at home or 3D‐printed at a low cost. Two 5 mm apertures were positioned on opposite sides of the cuvette chamber to accommodate interchangeable LEDs, enabling both 180° transmission geometry and 90° fluorescence/excitation geometry relative to the detector. For reproducibility, the optical path lengths were maintained such that the distance between the LED and cuvette was approximately 1 cm, and the cuvette‐to‐detector distance was also fixed at approximately 1 cm. All measurements were performed using standard plastic cuvettes with a 10‐mm optical path length. The detector was centrally aligned with respect to the cuvette to ensure consistent signal acquisition. Additionally, a 2‐mm slit aperture was placed between the cuvette and the detector to minimize the contribution of stray light and scattered radiation, thereby improving signal specificity and reducing background interference. Only wavelengths corresponding to the selected LED emission and sensor channel alignment were used for absorbance calculations. The sensor communicated with a microcontroller via the I^2^C protocol, and a custom Arduino‐based program was developed to control data acquisition and processing. The source code is shared on our GitHub repository [18]. Illumination intensity and switching of the LEDs were managed using an external LED controller. Measured data were transmitted and recorded through the serial monitor interface for subsequent analysis. Table 1 presents the cost of each component used for this project. The estimated component cost for one complete instrument is approximately US$50 (the exact price was US$47.62), excluding the optional breadboard, based on the per‐unit cost of components purchased in multiunit packages.

**FIGURE 2:**
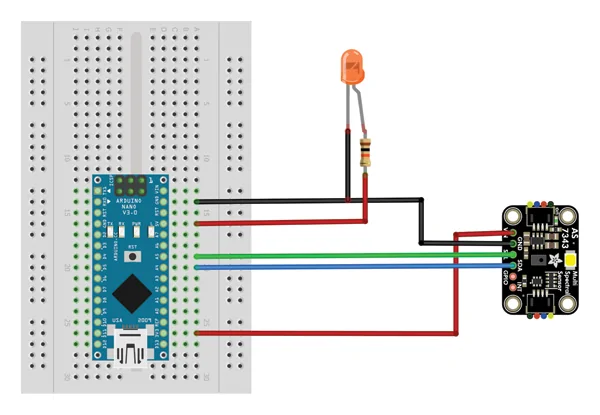
Detailed electrical diagram of do‐it‐yourself (DIY) spectrophotometer developed in this study.

**TABLE 1:** Cost details of each component used in this project.

S.N.	Item	Price (USD)	Cost per device (USD)
1	AS7343 sensor	21.95	21.95
2	Breadboard (optional)	8.00	—
3	Green LED (LED528EHP)	28.60 (5 pieces)	5.72
4	Red LED (LED630L)	14.62	14.62
5	Arduino Nano	15.99 (3 pieces)	5.33
Total			47.62

### 2.3. Data Acquisition and Measurement

Spectral measurements were performed using an AS7343 multichannel spectral sensor (SparkFun Electronics, USA) interfaced via the I^2^C protocol and controlled through a custom C++ program developed in the Arduino environment. The sensor was configured in 18‐channel AutoSMUX mode, with five wavelength channels selected at 515, 550, 555, 600, and 640 nm. The analog gain was set to 32*x*, and integration timing parameters were defined by ASTEP = 599 and ATIME = 199, yielding an effective integration time of approximately 100 ms according to the manufacturer’s equation:

(1) tint=ATIME+1∗ASTEP+1×2.78 μs.

A wait time of 30.6 µs was applied between measurements to ensure signal stability. Each reported value was based on a single sensor acquisition, without averaging multiple acquisitions. A blank (reference) measurement was first acquired after a 20 s equilibration period to obtain baseline intensity values (*I*_0_) for each wavelength. Subsequently, sample measurements were collected following a similar 20 s equilibration period, and corresponding intensity values (*I*) were recorded. Absorbance for each channel was calculated using the Beer–Lambert relationship:

(2) A=log10I0I,

with safeguards applied to return zero when either *I*_0_ or *I* was nonpositive to avoid undefined logarithmic values. The computed absorbance values were transmitted via serial communication at 115,200 baud, with measurements recorded at 20‐s intervals.

### 2.4. Conventional UV–Vis Spectrophotometer

To evaluate the performance of the LED spectrophotometer, the calibration and sample solutions described above were analyzed using a standard UV–Vis spectrophotometer (SpectraMax, Molecular Devices, CA, USA). Absorption spectra of CuSO_4_ and KMnO_4_ were recorded over the visible range to examine their optical absorption behavior. CuSO_4_ was scanned from 500 to 900 nm, while KMnO_4_ was scanned from 400 to 800 nm, revealing their characteristic absorption profiles (Figure S3). The absorbance of the solutions was measured at 640 nm for CuSO_4_ and 515 nm for KMnO_4_ solutions.

### 2.5. Fluorescence Measurement and Optical Configuration

Fluorescence measurements were performed using the same custom‐built optical sensing platform described for absorbance experiments, with a modification to the optical geometry to enable orthogonal excitation and emission detection. In the fluorescence configuration, the excitation LED and spectral sensor were positioned at approximately 90° relative to one another, with the sample cuvette located at the intersection of the excitation and detection paths. This configuration minimized direct transmission of excitation light into the detector and improved the collection of fluorescence emission from the sample solution. The modified arrangement was constructed using the same modular housing and electronic components employed in the absorbance measurements, allowing rapid conversion between absorbance and fluorescence modes without substantial hardware changes. A green LED with nominal emission centered at 528 nm was used as the excitation source for fluorescence experiments. Fluorescence emission was detected using the AS7343 multichannel spectral sensor, with signal monitoring focused primarily on the 600‐nm channel due to its overlap with the emission band of Rhodamine B.

A stock solution of Rhodamine B was first prepared at a concentration of 1 mM in deionized water. Intermediate and working solutions were subsequently prepared by serial dilutions with deionized water. Fluorescence calibration standards were prepared at concentrations of 0.5, 1, 2, 4, 6, 8, and 10 μM in a final volume of 4 mL. A blank solution consisting only of deionized water was used for background correction. Prior to each measurement, the blank solution was recorded to obtain background fluorescence and scattered excitation light contributions. Sample fluorescence signals were then collected after a 20‐s equilibration period to ensure optical stability and consistent positioning within the cuvette holder. Fluorescence intensity values were corrected by subtracting the blank signal according to *F*_corr_ = *F*_sample_ − *F*_blank_, where *F*_corr_ is the corrected fluorescence intensity, *F*_sample_ is the measured sample intensity, and *F*_blank_ is the blank intensity measured using deionized water. The analog gain was set to 64*x*.

### 2.6. Limit of Detection (LOD), Quantification, and Calibration Transfer

The analytical sensitivity and detection capability of the method are described by the LOD and limit of quantification (LOQ), which are defined as follows [19]:

(3) LOD=3.3σS,LOQ=10σS,

where *σ* represents the standard deviation of blank or low‐concentration measurements and *S* represents the slope of the calibration curve. The LOD and LOQ were determined separately for each analyte system using the corresponding DIY calibration parameters. To correct for systematic differences between instruments, a linear calibration transfer model was constructed relating the standard instrument response to the DIY instrument response. The model was expressed as follows [20]:

(4) ASTD=a+b∗ADIY,

where *A*_STD_ is the absorbance of the standard and *A*_DIY_ is the absorbance value measured from the DIY spectrophotometer, where *a* represents the intercept correction and *b* represents the scaling factor between instruments. Separate calibration transfer models were developed for both salts to evaluate whether instrument bias was analyte‐dependent. Regression parameters were obtained using least‐squares linear fitting.

### 2.7. Statistical Analysis

A two‐way analysis of variance (ANOVA) was performed to evaluate the effects of instrument type (DIY spectrophotometer and standard UV–Visible spectrophotometer) and analyte concentration on measured absorbance [21, 22]. The analysis was conducted separately for each analyte system, with instrument type considered as a fixed factor and concentration treated as a second fixed factor. Replicate measurements (*n* = 3 per concentration level) were included in the model to estimate experimental variability. The interaction term between instrument type and concentration was included to assess whether the difference between instruments depended on concentration. Prior to analysis, data were blank‐corrected and verified for linear calibration behavior. Normality of residuals was assumed based on replicate measurement structure, and homogeneity of variance was considered acceptable due to consistent replicate spread across concentration levels. Statistical significance was evaluated at a 95% confidence level (*α* = 0.05).

## 3. Results and Discussion

### 3.1. Validation of the Portable Spectrophotometer Using CuSO_4_ and KMnO_4_ Solutions

The analytical performance of the developed portable spectrophotometer was evaluated using aqueous solutions of copper (II) sulfate (CuSO_4_) and potassium permanganate (KMnO_4_), and compared with a conventional UV–Vis spectrophotometer to assess its suitability for quantitative absorbance analysis and validation of the Beer–Lambert law.

For CuSO_4_, measurements were performed using an external orange LED excitation source centered at approximately 630 nm, while absorbance values were monitored using the 640 nm detection channel of the AS7343 spectral sensor. Figure 3 shows the calibration relationship obtained between CuSO_4_ concentration and absorbance measured using the developed instrument. A positive linear trend was observed over the investigated concentration range (0–0.40 M), yielding the regression equation, *A* = 1.83*C* + 0.007 with a coefficient of determination of *R* ^2^ = 0.99. The observed linearity demonstrates that the portable system was capable of quantitatively detecting concentration‐dependent attenuation of transmitted light, consistent with Beer–Lambert behavior. The LOD and LOQ were calculated as 0.276 and 0.836 M, respectively. Minor deviations from ideal linearity at lower concentrations may arise from optical alignment, LED intensity fluctuations, ambient light interference, or the relatively broad spectral bandwidth of the LED source and sensor channels. For comparison, the same CuSO_4_ standards were analyzed using a conventional UV–Vis spectrophotometer operating at 640 nm. As shown in Figure 3, the standard instrument produced a highly linear calibration curve described by *A* = 1.644*C* + 0.005 with a coefficient of determination *R* ^2^ = 0.98.

**FIGURE 3:**
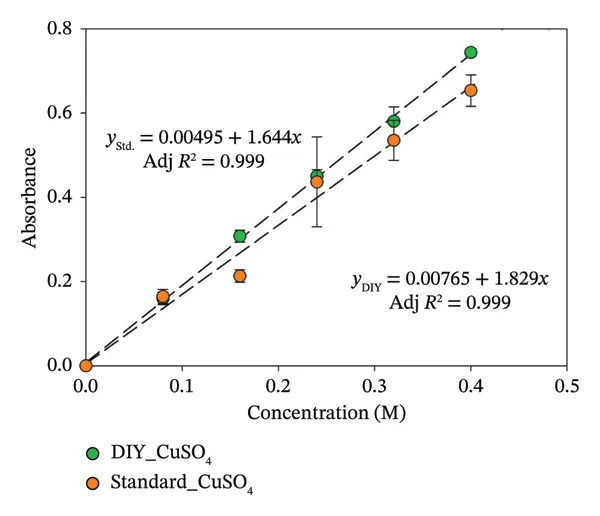
Absorbance of CuSO_4_ solutions measured using the DIY spectrophotometer (excitation: 630 nm; detection: 640 nm and a standard spectrophotometer (excitation/detection: 640 nm). Circles denote experimentally measured absorbance values, and the solid line represents the least‐squares fit. Data are presented as means and standard deviations of three replicate measurements.

The performance of the developed portable spectrophotometer was further evaluated using aqueous solutions of KMnO_4_ (0–10 mM), a strongly colored compound commonly used in spectrophotometric experiments due to its intense visible absorption characteristics. Measurements obtained using the custom‐built device were compared with those acquired using a conventional UV–Vis spectrophotometer to further assess the applicability of the system for quantitative analysis.

The temporal stability of the optical system was evaluated by monitoring the blank response at 640 nm over an extended period. As shown in Figure S4, the measured intensity exhibited a gradual decrease during the initial period of operation, followed by a relatively stable response. The blank response changed by approximately 5.3% over the full 450‐min stability test. However, a complete measurement run during the analytical experiments required approximately 5 min, substantially shorter than the duration of the stability test. Thus, the temporal change in LED output during an individual measurement run is expected to be small. In addition, dark measurements obtained during a separate 4‐h experiment produced signals near zero, indicating a negligible contribution from the detector dark response.

For the KMnO_4_ test, excitation was provided using a green LED centered at approximately 528 nm, while transmitted light intensity was monitored using the 515‐nm detection channel of the AS7343 spectral sensor. The resulting calibration plot exhibited a linear increase in absorbance with increasing KMnO_4_ concentration over the investigated range. Linear regression analysis produced the relationship *A* = 0.065*C* + 0.05 with a coefficient of determination *R* ^2^ = 0.97. The observed linearity confirms that the portable system was capable of quantitatively monitoring concentration‐dependent absorbance changes in KMnO_4_ solutions. The LOD and LOQ were calculated as 0.031 and 0.0.093 mM, respectively. The slightly lower correlation coefficient compared to the conventional spectrophotometer may be attributed to the broader emission bandwidth of the LED source, overlap between sensor detection channels, and minor variations in optical alignment and illumination intensity. The same KMnO_4_ solutions were subsequently analyzed using a conventional UV–Vis spectrophotometer at 515 nm. As shown in Figure 4, the standard instrument also demonstrated a strong linear relationship between concentration and absorbance, yielding *A* = 0.123*C* + 0.026 with *R* ^2^ = 0.98.

**FIGURE 4:**
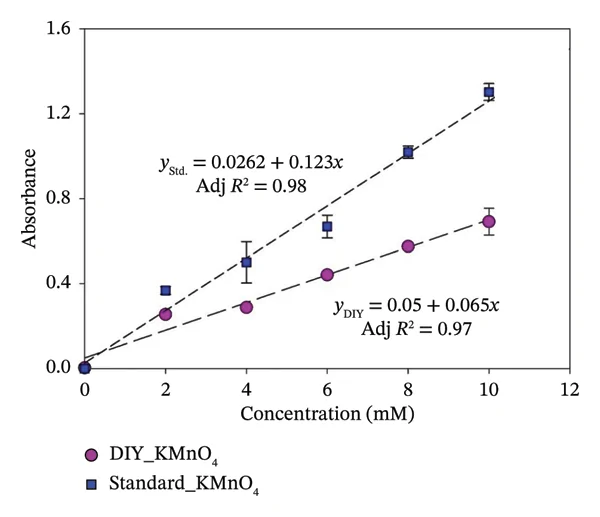
Absorbance of KMnO_4_ solutions measured using the DIY spectrophotometer (excitation: 528 nm; detection: 515 nm) and a standard spectrophotometer (excitation/emission: 515 nm). Circles denote experimentally measured absorbance values, and the solid line represents the least‐squares fit. Data are presented as the means and standard deviations of three replicate measurements.

Two‐way ANOVA was performed to evaluate the effects of concentration and instrument type (DIY spectrophotometer versus standard UV–Vis spectrophotometer) on absorbance responses for both CuSO_4_ and KMnO_4_ systems. For CuSO_4_, the analysis showed significant effects of concentration (*p* < 0.0001) and instrument type (*p* < 0.0001), while the interaction term was not significant (*p* > 0.05). This indicates that both instruments followed similar concentration‐dependent trends, differing mainly in overall response magnitude, which supports the validity of linear calibration transfer between the systems. In contrast, for KMnO_4_, two‐way ANOVA revealed significant effects of concentration (*F*(5, 24) = 404.95, *p* < 0.0001), instrument type (*F*(1, 24) = 343.75, *p* < 0.0001), and a highly significant interaction between concentration and instrument (*F*(5, 24) = 39.73, *p* < 0.0001). This indicates that, unlike CuSO_4_, the response of the DIY spectrophotometer deviated from the standard instrument in a concentration‐dependent manner, suggesting nonparallel calibration behavior and a nonuniform systematic distortion across the measurement range. Calibration transfer functions were established to relate the DIY spectrophotometer response to the standard UV–Vis instrument. For CuSO_4_, where no significant interaction was observed, a simple linear model was adequate, expressed as *A*_STD_ = −0.018 + 1.86*A*_DIY_, indicating consistent proportional and offset differences across the full concentration range. In contrast, for KMnO_4_, a significant interaction between concentration and instrument type indicated concentration‐dependent deviation, and the transfer was approximated by a global linear model *A*_STD_ = 0.032 + 1.72*A*_DIY_, although with increasing residual error at higher concentrations, reflecting nonparallel calibration behavior.

The commercial spectrophotometer produced improved linearity due to its monochromator‐based wavelength selection, narrower spectral bandwidth, and enhanced optical stability. Nevertheless, the portable device successfully reproduced the expected concentration‐dependent absorbance trend and provided calibration behavior consistent with Beer–Lambert principles. Differences in calibration slope between the portable and conventional instruments are likely associated with variations in excitation wavelength, detector spectral response, optical geometry, and path length characteristics. In particular, the use of a 528‐nm excitation source with detection at 515 nm in the portable device differs from the single‐wavelength monochromatic measurement employed by the commercial UV–Vis spectrophotometer. The KMnO_4_ experiments further highlight the flexibility of the developed platform, demonstrating that interchangeable LEDs and selectable sensor channels can be adapted for multiple analytes and wavelength regions.

### 3.2. Fluorescence Detection of Rhodamine B Using the DIY Spectrophotometer

The fluorescence capability of the portable spectrophotometric platform was evaluated using Rhodamine B as a model fluorophore. To enable fluorescence measurements, the optical geometry of the system was modified from the transmission‐based absorbance configuration to an orthogonal arrangement in which the excitation LED and spectral detector were positioned at approximately 90° relative to one another [23]. This configuration reduced direct excitation light reaching the detector and improved sensitivity toward fluorescence emission from the sample solution. A 528‐nm LED was employed as the excitation source, while fluorescence emission was monitored primarily using the 600‐nm channel of the AS7343 spectral sensor [24]. Background‐corrected fluorescence intensity increased with increasing Rhodamine B concentration over the evaluated concentration range of 0–10 μM, demonstrating successful fluorescence detection using the low‐cost sensing platform. The fluorescence response exhibited a linear relationship over the concentration range of 0–4 μM, which was defined as the linear range, with the calibration equation *F* = 8.883*C* + 0.548 and a coefficient of determination of *R* ^2^ = 0.99, where F represents the corrected fluorescence intensity, and C is the Rhodamine B concentration in μM (Figure 5). The strong linearity observed within this concentration range indicates that the system is capable of semiquantitative fluorescence analysis. The LOD and LOQ were calculated as 0.218 μM and 0.659 μM, respectively, demonstrating that the method can detect submicromolar concentrations of Rhodamine B with good sensitivity. The relatively low LOD reflects the strong fluorescence emission characteristics of Rhodamine B and favorable signal‐to‐noise performance at low concentrations.

**FIGURE 5:**
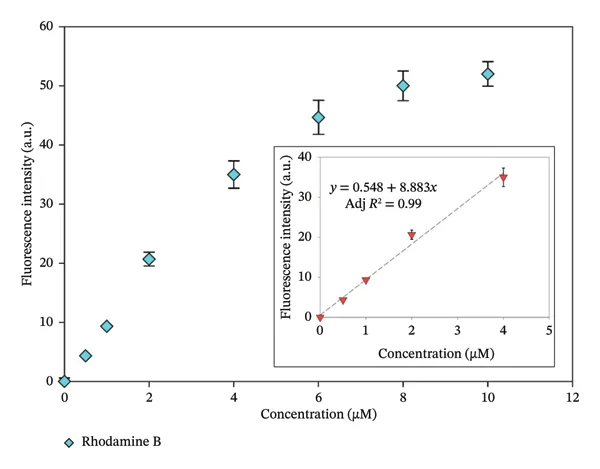
Fluorescence response of Rhodamine B measured using the portable spectrophotometric platform with 528‐nm LED excitation and detection at 600 nm. Data are presented as means and standard deviations of three replicate measurements. Fluorescence intensity increased with increasing Rhodamine B concentration over the investigated range of 0.5–10 μM. The inset shows the linear calibration region between 0 and 4 μM. Deviation from linearity at higher concentrations is attributed to fluorescence saturation and inner‐filter effects.

At concentrations above approximately 4 μM, deviations from linearity and gradual signal saturation were observed. This behavior is attributed to fluorescence self‐quenching, inner‐filter effects, and limitations associated with detector response at higher fluorophore concentrations. Nevertheless, measurable fluorescence signals were obtained up to 10 μM, defining the practical response range of the device for fluorescence measurements and demonstrations. Thus, the evaluated concentration range and practical response range were 0–10 μM, whereas 0–4 μM represented the linear range for quantitative analysis. The results demonstrate that the modular spectrophotometer can be adapted for both absorbance and fluorescence measurements using only minor modifications to the optical configuration. The current method can also be implemented for colorimetric detection in various low‐cost applications [25, 26].

## 4. Conclusion

This study presents a portable, low‐cost, and modular spectrophotometric platform for wavelength‐selective optical measurements. The system integrates interchangeable visible‐light LEDs, a compact multichannel spectral sensor, and microcontroller‐based data acquisition, enabling a compact implementation of absorbance and fluorescence spectroscopy with improved portability and accessibility. The instrument was validated using aqueous solutions of CuSO_4_ and KMnO_4_. The resulting calibration curves exhibited Beer–Lambert behavior and showed good agreement with measurements obtained using a conventional UV–Vis spectrophotometer. Strong linear responses were observed across concentration ranges, demonstrating reliable analytical performance. Two‐way ANOVA revealed a significant effect of both concentration and instrument type (*p* < 0.05), while no significant interaction was observed, indicating parallel calibration behavior between the DIY and standard instruments. However, systematic differences in calibration slopes were observed, confirming instrument‐dependent sensitivity variation. These differences were effectively described using linear calibration transfer models, demonstrating that DIY measurements can be reliably converted to standard‐equivalent values using analyte‐specific correction factors. The platform was further adapted for fluorescence measurements through reconfiguration of the optical geometry to a 90° excitation–emission setup. Using Rhodamine B as a model fluorophore, the system exhibited concentration‐dependent fluorescence response with good linearity in the low micromolar range, confirming its suitability for fluorescence‐based analysis. The results demonstrate that the proposed system provides a versatile and cost‐effective alternative for basic spectroscopic measurements, supporting both absorbance and fluorescence modalities within a unified, modular architecture. The analytical evaluation in this study was conducted using model aqueous solutions; validation with real and more complex sample matrices remains an important direction for future work.

The current study has several limitations, including the evaluation of a limited number of model analytes using simple aqueous solutions and the absence of validation with real samples. In addition, manual optical alignment is required when configuring the system for different measurement modes, and the accessible measurement wavelengths are dependent on the spectral characteristics of the selected LEDs and sensor channels. Future work will focus on expanding the range of analytes and real‐sample applications, while improving optical alignment and wavelength flexibility.

## Author Contributions

Sanam Pudasaini, Beni B. Dangi, and Vijay Singh Chhetri designed the work; Sanam Pudasaini and Rajesh Singh Rathore performed the experimental work; Sanam Pudasaini and Prakash Dangal analyzed the data; Vijay Singh Chhetri and Beni B. Dangi obtained funding; and all authors contributed to the writing and editing of the manuscript.

## Funding

This work was supported by the NASA through grant no. 80NSSC23M0050 and 1890 Center of Excellence for Student Success and Workforce Development, 2021‐38427‐34837.

## Conflicts of Interest

The authors declare no conflicts of interest.

## Supporting Information

Additional supporting information can be found online in the Supporting Information section.

## Supporting information


**Supporting Information** Additional experimental details.

## Data Availability

The data that support the plots within this paper and other findings of this study are available from the corresponding author upon reasonable request.
