# Supplementary material for: A Portable, Low‐Cost Spectrophotometer for Absorbance and Selected‐Wavelength Fluorescence Measurements
Source: J Anal Methods Chem. 2026 Oct 28;2026:9371485. doi: 10.1155/jamc/9371485 (PMC13717223; doi:10.1155/jamc/9371485)
Supplement: Supplementary file 1 [file JAMC-2026-9371485-s001.zip › SI_R1.docx]

Supporting Information

A Portable, Low-Cost Spectrophotometer for Absorbance and Selected-Wavelength Fluorescence Measurements

Sanam Pudasaini,^1^ Prakash Dangal,^2^ Rajesh Singh Rathore,^3^ Beni B. Dangi,^1*^ and Vijay Singh Chhetri ^2*^

^1^Department of Chemistry, Florida Agricultural and Mechanical University, Tallahassee, FL, 32307, USA

^2^ Food Science Program, College of Agriculture and Food Sciences, Florida Agricultural and Mechanical University, Tallahassee, FL, 32307, USA

^3^ School of the Environment, Florida Agricultural and Mechanical University, Tallahassee, FL, 32307, USA

*[vijay.chhetri@famu.edu](mailto:vijay.chhetri@FAMU.EDU) , *[beni.dangi@famu.edu](mailto:beni.dangi@famu.edu)

**Figure S1:** Spectral Intensity Distribution for LED528EHP obtained from the manufacturer's specification sheet.

**Figure S2:** Spectral Intensity Distribution for LED630L obtained from the manufacturer's specification sheet.

**Figure S3:** Absorption spectra of (A) copper (II) sulfate (CuSO₄) recorded over the wavelength range 500-900 nm, and (B) potassium permanganate (KMnO₄) recorded over 400-800 nm.

**Table S1:** Two-way ANOVA results for CuSO_4_ comparing absorbance responses between the DIY and standard spectrophotometers as a function of instrument type and concentration.

| *Source of Variation* | *SS* | *df* | *MS* | *F* | *P-value* | *F crit* |
| --- | --- | --- | --- | --- | --- | --- |
| Sample | 2.033470472 | 5 | 0.406694 | 278.9238 | 1.947E-20 | 2.6206541 |
| Columns | 0.014200694 | 1 | 0.014201 | 9.739289 | 0.0046495 | 4.2596773 |
| Interaction | 0.014647806 | 5 | 0.00293 | 2.009186 | 0.1135747 | 2.6206541 |
| Within | 0.034994 | 24 | 0.001458 |  |  |  |
|  |  |  |  |  |  |  |
| Total | 2.097312972 | 35 |  |  |  |  |

Table S2: Two-way ANOVA results for KMnO_4_ comparing absorbance responses between the DIY and standard spectrophotometers as a function of instrument type and concentration.

| *Source of Variation* | *SS* | *df* | *MS* | *F* | *P-value* | *F crit* |
| --- | --- | --- | --- | --- | --- | --- |
| Sample | 3.797373 | 5 | 0.759475 | 403.2728 | 2.5E-22 | 2.620654 |
| Columns | 0.643739 | 1 | 0.643739 | 341.8183 | 1.06E-15 | 4.259677 |
| Interaction | 0.376276 | 5 | 0.075255 | 39.95964 | 7.18E-11 | 2.620654 |
| Within | 0.045199 | 24 | 0.001883 |  |  |  |
|  |  |  |  |  |  |  |
| Total | 4.862586 | 35 |  |  |  |  |

| Time (mins) | Average intensity (counts) |
| --- | --- |
| 0 | 1228.3 |
| 30 | 1213 |
| 60 | 1201.5 |
| 90 | 1194.2 |
| 120 | 1187.9 |
| 150 | 1181.7 |
| 180 | 1176.6 |
| 210 | 1170.7 |
| 240 | 1160.5 |
| 270 | 1163.7 |
| 300 | 1167.3 |
| 330 | 1168.7 |
| 360 | 1167.7 |
| 390 | 1161.8 |
| 420 | 1161.8 |
| 450 | 1162.8 |

**Figure S4:** Temporal stability of the LED-based optical system evaluated using a blank at 640 nm. Each data point represents the mean of 10 replicate measurements, with error bars indicating the corresponding variability. The blank intensity exhibited a gradual decrease during the initial period followed by a relatively stable response during extended operation.

**Table S2:** Comparison of the proposed portable spectrophotometer with representative previously reported portable spectrophotometric systems.

| System | Cost | Dimensions | Spectral range | Resolution | Channels | Modes | Applications |
| --- | --- | --- | --- | --- | --- | --- | --- |
| This work | $50 | 6.5 in x 2.1 in | 380–1000 nm | Discrete multispectral channels | 1 | Absorbance, fluorescence | Chemical/biological analysis |
| Ref. [1] | $25 | 5 in. × 10 in. | ~400–700 nm | ~20 nm | 1 | Absorbance | Educational/quantitative analysis |
| Ref. [2] | $105 | 6.5 in x 2.1 in (+ Raspberry PI) | 450–640 nm | ~10 nm | 1 | Absorbance | Undergraduate laboratory, methylene blue analysis |
| Ref. [3] | $100 | - | Visible / UV–vis | X nm | Full spectrum | Absorbance, fluorescence, AES | KMnO₄, fluorescein, atomic emission |
| Ref. [4] | $9 | - | 585–590 nm | Single wavelength | 1 | Absorbance | Fe²⁺ analysis in water |
| Ref. [5] | $14 | - | Visible | - | Full spectrum | Absorbance + fluorescence | Dye spectroscopy, Beer–Lambert law, Stokes shift, education |
| Ref. [6] | - | - | ~430 and ~630 nm | - | 1 | Absorbance | p-Nitroaniline and Brilliant Blue analysis |
| Ref. [7] | - | - | UV–IR demonstrated (~357–871 nm) | ~2 nm FWHM | Full spectrum | Spectral analysis / absorbance-type measurements | Chemical analysis, environmental monitoring, astronomy, biomedical research, education |
| Ref. [8] | $2,800 total | - | 350–700 nm | Up to 2.0 nm | Full spectrum | Fluorescence / emission | Turmeric, riboflavin, CdSe/ZnS quantum dots; undergraduate spectroscopy |
| Ref. [9] | $50 | 5 × 5 × 8 cm | 405–690 nm | ~20 nm | 8 visible channels | Absorbance + fluorescence | Cu(II), BSA, CT-DNA; colorimetric and fluorescence analysis |
| Ref. [10] | $35–40 | - | ~490–710 nm demonstrated; UV fluorescence possible >300 nm | ~0.6 nm (R ≈ 900 at 532 nm) | Full spectrum / camera-based | Absorbance + fluorescence + emission | KMnO₄ absorption, quinine fluorescence, emission spectroscopy |

**References**

1.	Albert, D.R., M.A. Todt, and H.F. Davis, *A low-cost quantitative absorption spectrophotometer.* Journal of Chemical Education, 2012. **89**(11): p. 1432-1435.

2.	Bougot-Robin, K., et al., *Optimization and design of an absorbance spectrometer controlled using a Raspberry Pi to improve analytical skills.* Journal of Chemical Education, 2016. **93**(7): p. 1232-1240.

3.	DelMonache, G., et al., *Multipurpose DIY spectrophotometer for teaching analytical chemistry*. 2025, ACS Publications.

4.	Place, B.J., *Activity analysis of iron in water using a simple LED spectrophotometer.* Journal of Chemical Education, 2019. **96**(4): p. 714-719.

5.	Kolesnichenko, P.V., et al., *Viking spectrophotometer: a home-built, simple, and cost-efficient absorption and fluorescence spectrophotometer for education in chemistry.* Journal of Chemical Education, 2023. **100**(3): p. 1128-1137.

6.	Hosker, B.S., *Demonstrating principles of spectrophotometry by constructing a simple, low-cost, functional spectrophotometer utilizing the light sensor on a smartphone.* Journal of Chemical Education, 2018. **95**(1): p. 178-181.

7.	Patil, V.B. and P.S. Khodade, *Development and applications of an affordable DIY optical spectrometer using a webcam.* Journal of Optics, 2025: p. 1-4.

8.	Dangi, B.B., et al., *Molecular Emission Spectroscopy: A Laboratory Experiment for Undergraduate Students in Chemical Sciences.* Journal of Chemical Education, 2025. **102**(2): p. 754-759.

9.	Yu, Z., et al., *An open-source handheld spectrometer for colorimetric and fluorescence analyses.* Spectrochimica Acta Part A: Molecular and Biomolecular Spectroscopy, 2023. **287**: p. 122072.

10.	Bruininks, B. and L.B. Juurlink, *An inexpensive 3D printed periscope-type smartphone-based spectrophotometer for emission, absorption, and fluorescence spectrometry.* Journal of Chemical Education, 2022. **99**(5): p. 2168-2174.
