# Supplementary material for: A Portable, Low‐Cost Spectrophotometer for Absorbance and Selected‐Wavelength Fluorescence Measurements
Source: J Anal Methods Chem. 2026 Oct 28;2026:9371485. doi: 10.1155/jamc/9371485 (PMC13717223; doi:10.1155/jamc/9371485)
Supplement: Supplementary file 1 [file JAMC-2026-9371485-s001.zip › SI_V2.docx]

Supporting Information

A Portable, Low-Cost Spectrophotometer for Absorption and Fluorescence Measurements

Sanam Pudasaini,^1^ Prakash Dangal,^2^ Rajesh Singh Rathore,^3^ Beni B. Dangi,^1*^ and Vijay Singh Chhetri ^2*^

^1^Department of Chemistry, Florida Agricultural and Mechanical University, Tallahassee FL, 32307, USA

^2^ Food Science Program, College of Agriculture and Food Sciences, Florida Agricultural and Mechanical University, Tallahassee FL, 32307, USA

^3^ School of the Environment, Florida Agricultural and Mechanical University, Tallahassee FL, 32307, USA

*[vijay.chhetri@famu.edu](mailto:vijay.chhetri@FAMU.EDU) , *[beni.dangi@famu.edu](mailto:beni.dangi@famu.edu)

**Figure S1:** Spectral Intensity Distribution for LED528EHP obtained from manufacturer specification sheet.

**Figure S2:** Spectral Intensity Distribution for LED630L obtained from manufacturer specification sheet.

**Figure S3:** Absorption spectra of (A) copper (II) sulfate (CuSO₄) recorded over the wavelength range 500-900 nm, and (B) potassium permanganate (KMnO₄) recorded over 400-800 nm.
